# *Operando* time-gated Raman spectroscopy of solid catalysts†

**DOI:** 10.1039/d3cy00967j

**Published:** 2023-09-07

**Authors:** Robin Vogel, P. Tim Prins, Freddy T. Rabouw, Bert M. Weckhuysen

**Affiliations:** a Inorganic Chemistry and Catalysis Group, Institute for Sustainable and Circular Chemistry and Debye Institute for Nanomaterials Science, Utrecht University Universiteitsweg 99 3584 CG Utrecht The Netherlands b.m.weckhuysen@uu.nl; b Soft Condensed Matter Group, Debye Institute for Nanomaterials Science, Utrecht University Princetonplein 1 3584 CC Utrecht The Netherlands

## Abstract

*Operando* Raman spectroscopy is a powerful analytical tool to provide new insights in the working and deactivation principles of solid catalysts. Intense fluorescence can obscure Raman spectra to the extent that they become uninterpretable. Time-gated Raman spectroscopy, based on pulsed excitation and time-gated detection, suppresses background fluorescence based on its slower time dynamics compared to Raman scattering. In this work, we demonstrate and quantify the benefit of time gating for *operando* Raman spectroscopy, using the propane dehydrogenation reaction over Pt–Sn-based catalyst materials as a case study. Experimental time-gated Raman spectroscopy data are fitted to a time-trace model that is used to optimize time gating for the maximum signal-to-background-noise ratio. Time-gated Raman spectra of a spent propane dehydrogenation catalyst material show lower background fluorescence compared to the time-integrated Raman spectra counterparts. Simultaneous *operando* time-gated and time-integrated Raman spectroscopy experiments demonstrate the benefit of time gating to obtain more distinct Raman features, especially in the early coking stages where spectra are dominated by background fluorescence.

## Abstract 2

Simultaneous time-gated and time-integrated Raman spectroscopy quantifies the benefit of time gating to obtain clearer Raman features in the early coking stages of propane dehydrogenation, when the spectra are dominated by background fluorescence.

## Graphical abstract

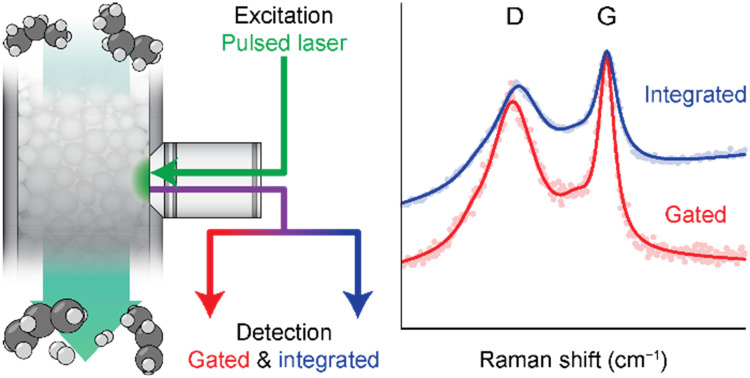

## Introduction

*Operando* Raman spectroscopy is a powerful analytical tool to provide new or improved insights in the working and deactivation principles of solid catalysts at work. Spectroscopic information can be interpreted together with online product analysis to couple physicochemical events occurring on the surface of the solid catalyst with its activity, selectivity, and stability.^1–3^*Operando* Raman spectroscopy revealed, for example, a correlation between the formation of carbon deposits and catalyst deactivation during *e.g.*, Fischer–Tropsch synthesis (FTS) or propane dehydrogenation (PDH), which are both industrially relevant catalytic processes.^4–6^

Raman scattering is an intrinsically weak process, so that its spectral features are sometimes overshadowed by other signals reaching the detector. Intense background signals may arise from sample fluorescence, ambient light, or black body radiation. In practice, Raman spectra processing often includes a background subtraction. This can, however, be challenging if the background signal has a particular spectral shape. Moreover, random noise cannot be subtracted and becomes higher with increasing background intensity.^7^ At some point, the signal-to-noise ratio (SNR) of Raman bands can become so low that the bands are indistinguishable from the background, resulting in spectra that are uninterpretable.^4,5^

Several techniques have been developed to improve the quality of Raman spectra. Common strategies involve the enhancement of Raman signal. For instance, nanostructured plasmonic surfaces or plasmonic nanoparticles can boost the Raman signal intensity, but the stability of such nanostructures is still limited, especially at elevated temperatures, thereby hampering their applicability for *operando* spectroscopy purposes.^8–11^ The background fluorescence can also be reduced by shifting to longer laser excitation wavelengths. This makes electronic transitions in organic molecules, resulting in fluorescence, less likely. Shifting to lower excitation energy however lowers the chance of a Raman scattering event.^12^ Alternatively, UV lasers are used as excitation sources to avoid background fluorescence. In this case, background fluorescence induced by the excitation of aromatic molecules appears at longer wavelengths than the Raman features.^13^ This principle has been applied for the study of coke formation during the methanol-to-olefins (MTO) reaction.^14,15^

Time-gated Raman spectroscopy offers improved spectral quality over conventional Raman spectroscopy. It suppresses background fluorescence based on its slower time dynamics compared to Raman scattering. Raman scattering occurs instantaneously after excitation, whereas fluorescence of organic molecules typically exhibits a lifetime on the ns scale.^16^ By time gating the detection, background fluorescence and Poisson noise on it are reduced and the spectral quality is improved. Fluorescence can be rejected using an optical Kerr gate^17,18^ or using detectors with a high time resolution, typically complementary metal-oxide-semiconductor single-photon avalanche diodes (CMOS-SPAD).^12,19–21^

Time-gated Raman spectroscopy is applied in a wide variety of fields yielding insightful results. Kerr-gated Raman spectroscopy (KG-RS) has been applied for the study of carbonaceous species on solid catalysts, including the formation of coke species during the MTO reaction^22^ and carbon deposits on other strongly fluorescent zeolite-based catalyst materials,^23,24^ as well as in battery research.^25,26^ CMOS-SPAD-based time-gated Raman spectroscopy was used for numerous different type of samples, including biological samples and pharmaceuticals,^27–31^ rare-earth-element containing rock samples,^32^ high-temperature melts,^33^ and pickle liquor.^34^

Precisely assessing the beneficial effect of time gating on spectral quality remains challenging. In some studies, time-gated Raman spectroscopy was compared with conventional Raman spectroscopy using a qualitative approach.^27,28,32^ A few studies quantitatively assed the effect of time gating based on CMOS-SPAD detectors.^35,36^ Kekkonen *et al.* reported a 4.4–8.8 fold higher signal-to-peak-to-peak-noise ratio (SNRpp) in time-gated compared to conventional Raman spectroscopy on a tooth sample. Kotula *et al.* used the ratio of background-corrected Raman signal to the standard deviation in the background (SBNR) as a quality descriptor and reported an 8–68-fold increase in spectral quality from recovered plastics. However, switching between two spectroscopy modes inevitably results in working with two spectrometers with different designs and different optical paths. A quality descriptor that depends on photon-count rates, such as the SNR, tests not only the effect of time gating but also the detection efficiency of the experimental setup.^35,36^

In this work, we demonstrate and quantify the benefit of CMOS-SPAD-based time gating for *operando* Raman spectroscopy for studying solid catalysts at work. We follow a Raman-microscopy-based approach for the *ex situ* analysis of spent PDH catalysts. Based on an analysis of the time dynamics of Raman scattering and background fluorescence upon pulsed laser excitation, we select the time gate for an optimal signal-to-background-noise ratio (SBNR). Optimally time-gated Raman spectra are compared to time-integrated Raman spectra recorded with a pulsed laser and a CCD detector. The lessons learned are applied in an *operando* simultaneous time-gated and time-integrated Raman spectroscopy experiment of a PDH catalyst material in a laboratory-scale reactor. The results illustrate the urgency to consider sample heterogeneity for analyses of the catalyst materials under study and highlight that time gating lowers the background fluorescence, to make Raman features better distinguishable.

## Results and discussion

### Working principle of time-gated Raman spectroscopy

**Fig. 1:**
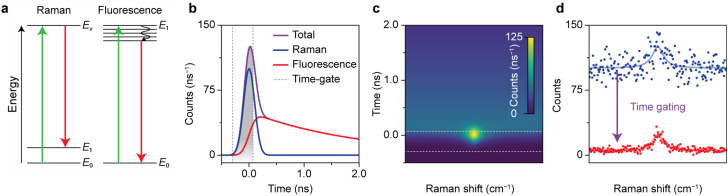
Working principle of time-gated Raman spectroscopy. (a) Schematic representation of Stokes Raman scattering and fluorescence. (b) Simulated dynamics of the signal recorded following pulsed laser excitation, constituting Raman scattering (blue) and fluorescence (red). As Raman scattering occurs instantaneously, it has the shape of the Gaussian laser pulse (FWHM = 0.235 ns, 25 integrated counts). Fluorescence dynamics has the time shape of the Gaussian laser pulse convoluted with exponential decay with 2 ns lifetime (100 integrated counts). (c) Simulated heatmap of a Raman band with a typical Lorentzian line shape and a broadband background fluorescence (with the same characteristics as in panel b). (d) Simulated time-gated and time-integrated spectrum of panel c, with additional Poisson noise. The application of the optimal time gate increases the spectral quality in terms of SBNR from 2.5 to 9.4.

Fig. 1 shows how the optimization of time-gated Raman spectroscopy and its effectivity can be assessed with a time-trace model. Time-gated Raman spectroscopy was developed to enhance the quality of Raman spectra by rejecting background fluorescence and resulting Poisson noise based on its slower time dynamics. The competing events are schematically presented in Fig. 1a. Raman scattering occurs instantaneously, when an analyte undergoes a transition to a virtual excited state and falls back to a vibrationally excited state. An incident photon can also induce fluorescence, due to an electronic transition followed by internal relaxation to the lowest excited state and radiative decay back to the ground state. The characteristic lifetime of the emission depends on the type of transition, but is typically of the order of a few ns for organic molecules.^16^ Pulsed excitation is used to discriminate between the two events based on their differences in time dynamics. Both signals are convoluted with the instrument response function (IRF), which we approximate as a Gaussian. The resulting time traces are shown in Fig. 1b.

Choosing the proper quality descriptor for spectral quality is key for drawing conclusions about the effectiveness of time gating.^35,36^ In this work, we have selected two quality descriptors. For the signal-to-background-noise ratio (SBNR), the signal is defined as the amplitude of a Raman band compared to the standard deviation on the background signal, calculated using the noise characteristics of the detectors. The model used in this section assumes Poisson noise, so that the background noise equals the square root of the background counts. The SBNR can be regarded a quantitative descriptor for the spectrum that illustrates the Poisson noise suppression due to time gating. A drawback of this parameter is that it is not intrinsic, but depends on the integration time and the efficiency of the detector system. In addition, we have opted for the Raman-to-total ratio (RTR): the ratio of the amplitude of the Raman band above the background to the total signal in the Raman band. This parameter describes the shape of the spectrum and is independent of the number of counts.

The time-trace model can predict which time gating settings yield the optimal spectrum.^19^Fig. 1c shows the simulated time-dependent signal of Raman scattering with a Lorentzian line shape plus a spectrally flat fluorescent background. The simulated time-integrated and optimally time-gated spectra are shown in Fig. 1d. Time gating suppresses the Raman signal slightly, while the background and the associated background noise is suppressed considerably. As a result, the time-gated Raman spectrum exhibits a band that is better distinguishable. For the case with input parameters shown in Fig. 1, the time gate that yields the highest SBNR opens at the rise of the signal, closes at 0.07 ns, and has a width of 0.37 ns, 1.57 times the FWHM of the laser pulse. This confirms previous studies that found an optimal time-gate width of 1.5–2 times the FWHM of the laser pulse.^19,42^ Time gating reduces the background by 96% and the background noise by 81%, while the Raman signal decreases by 27%. This improves the SBNR of the time-gated spectrum to 9.4, compared to 2.5 for the time-integrated spectrum.

### Optimization of time-gated Raman spectroscopy for catalysis research

**Fig. 2:**
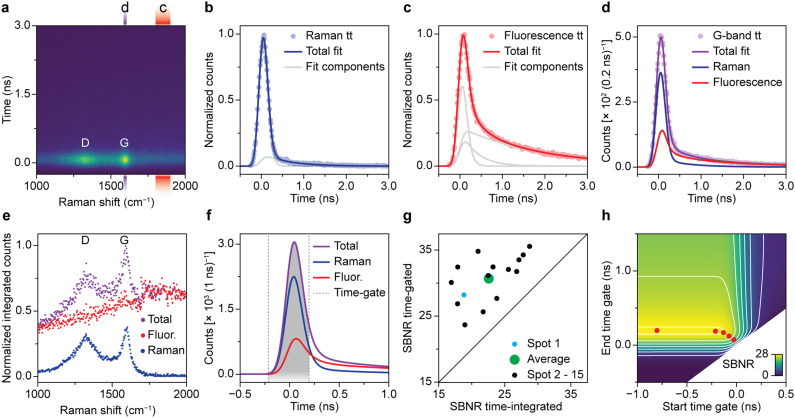
Optimization time-gated Raman spectroscopy for measuring coked Pt–Sn-based propane dehydrogenation (PDH) catalyst materials. (a) Experimental time-gated Raman spectroscopy data (200 ps time-gate width) of a spent 0.5 wt% Pt 1.5 wt% Sn/Al_2_O_3_ PDH catalyst, spot 1. The red and purple rectangles highlight spectral ranges of the background fluorescence and G-band, respectively. (b) Normalized time trace of the Raman scattering at 515–525 cm^−1^ in a reference measurement on a silicon wafer fitted with two E*G*B components. (c) Normalized time trace of the background fluorescence (1800–1900 cm^−1^) of spot 1 fitted with three E*G*B components. (d) Time trace at the spectral range of the G-band (1590–1610 cm^−1^), measured at spot 1 and fitted with a linear combination of the Raman and fluorescence time-trace fit. (e) Normalized time-integrated counts of fluorescence, Raman, and total (Raman + fluorescence) contributions as a function of Raman shift, obtained by linear-combination fitting of the time dynamics of panel a. (f) The deconvolved (E*G*B → E*G) time dynamics at the spectral range of the G-band of panel d. The time gate for optimal SBNR is highlighted in gray. (g) SBNR of the G-band for the time-integrated signal *versus* the optimally time-gated signal for 15 spots. (h) The SBNR of the G-band for spot 1 as a function of the start and end of the time gate. The red dots mark the optimal positions for time-gate widths of 0.1, 0.2, 0.3, 0.4, and 1 ns.

Fig. 2 quantifies the benefit of time gating for Raman spectroscopy on a spent Pt–Sn-based PDH catalyst material. Quantitative assessment of time gating facilitates both its validation and optimization for *operando* spectroscopy experiments. A typical time-resolved Raman spectrum recorded on spent and coked catalyst material is shown in Fig. 2a. Overall, the time dynamics at all Raman shift values is dominated by a fast rise in counts that peaks at 0 ns. The intensity at *t* = 0 ns is highest at 1320 and 1600 cm^−1^. These peaks, associated with coke deposits, originate from the Raman D-band associated with ring breathing modes of the edges and defects of graphitic sheets and the Raman G-band due to ring breathing modes of graphitic sheets, respectively.^5^ The ratio between de bands as well as the exact position and width can be used to deduce the chemical nature of the carbon deposits.^5^ We further observe spectrally broad background signal, which decreases fast (sub ns) and has a low-intensity tail that decays on the timescale of a few ns. This is assigned to fluorescence of carbonaceous species on the surface of the spent catalyst material.

Fig. 2b–d quantifies the contribution of Raman scattering and fluorescence at 1590–1610 cm^−1^, *i.e.*, the area of the G-band. To determine the dynamics of solely Raman scattering, we use a reference measurement on a crystalline silicon wafer (Fig. 2b), while the dynamics of fluorescence are obtained from the spent catalyst at 1800–1900 cm^−1^ (Fig. 2c). We fit the two reference measurements of Fig. 2b and c with a linear combination of two or three time components, respectively. Each component is the convolution of exponential decay with a Gaussian due to the laser pulse shape and a box function due to the time gating: E*G*B (Fig. S3†). Each component has a separate lifetime value, which we optimize during the fitting procedure. The fastest components, due to Raman scattering or sub-ns fluorescence, have a lifetime approaching 0 ns. Next, the signal dynamics from the spent catalyst at 1590–1610 cm^−1^ (Fig. 2d) are fitted to a linear combination of Raman scattering and fluorescence, based on our knowledge of the time dynamics of the two phenomena. Not surprisingly, we observe that the signal at *t* = 0 is dominated by Raman scattering, while the tail is mostly due to fluorescence.

We have applied the fit procedure to the time dynamics at all Raman shift values and determined the time-integrated contributions of the Raman scattering and fluorescence. Fig. 2e shows that this procedure separates the Raman spectrum with distinct peaks and low background (blue) from a broad background (red). Hence, although most of the fluorescence is sub ns, the time-trace fit procedure can disentangle both processes and produces clean spectra.

Based on our understanding of the time dynamics of Raman and fluorescence signals, we can determine the optimal settings for a time-gated experiment. The full time-resolved experiment of Fig. 2a allows us to separate clean Raman and fluorescence spectra (Fig. 2e), but the recording of this dataset is time consuming (185 s). Recording only the part of the signal pulse that yields the Raman spectrum with the highest quality only requires a fraction of that time (*e.g.*, 1.87 s, 4 of the 396 spectra that make up the time-resolved dataset) and is therefore preferable for many applications, such as an *operando* spectroscopy investigation of solid catalysts. Fig. 2f shows how we can use the results of the fit of the G-band as the basis for a time-trace model. We deconvolve the time-gate width of 200 ps from the data of Fig. 2a–e, yielding the trace of G*E shown in Fig. 2f and S3.† This model can be used to establish which start and end points of the time gate, *t*_1_ and *t*_2_, yield the optimal Raman spectrum. We define the SBNR as:
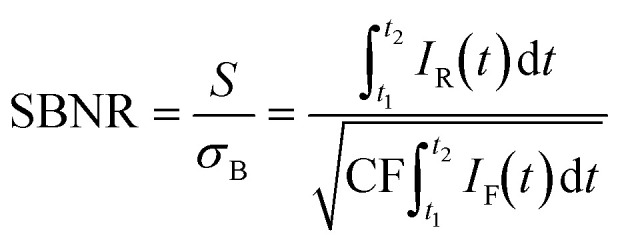
where *S* is the Raman signal in the G-band obtained by integrating the Raman intensity *I*_R_. *σ*_B_ is the Poisson noise on the fluorescent background intensity *I*_F_. The counts are presented after a sensitivity correction, that effectively multiplied the counts in the studied region of the spectrum with 1.5. The calculated noise is corrected for this modulation with the correction factor CF (Fig. S2†). The optimal SBNR of 28.3 is achieved for start and end points *t*_1_ = −0.214 ns and *t*_2_ = 0.202 ns of the time gate. This is better than the SBNR of 18.8 without time gating. Yet, the sub-ns component of the fluorescence makes complete suppression of background noise by time gating difficult in this case.

Fig. 2g illustrates that the improvement of SBNR upon application of a time gate varies considerably from spot to spot. We followed the described time-trace fit procedure for 15 spots on the same sample and tested the reproducibility of the method. The optimally time-gated SBNR ratio is plotted against the time-integrated SBNR. All datapoints are located above the diagonal, indicating that for all studied spots, the spectral quality improves when a time gate is applied. The extent to which the spectral quality improves varies strongly and hints towards heterogeneity in the sample.

We have used the deconvolved time trace of the G-band of spot 1 (Fig. 2f) to calculate the SBNR as a function of start and end position of the time gate, *i.e.*, *t*_1_ and *t*_2_. Fig. 2g shows the result. The map illustrates that the time gate should open at the rise of the signal and be long enough to collect sufficient Raman photons. The time-gated spectrometer in practice allows for the application of a finite number of gate widths: 0.1, 0.2, 0.3, 0.4, or 1.0 ns. The gates can be arbitrarily shifted in time with respect to the laser pulse. The red dots illustrate the optimal opening and closing positions for the available time gates. The optimal SBNR increases with increasing gate widths and levels off at 0.4 ns. Using the 1.0 ns time gate that opens long before the arrival of the laser pulse will yield a slightly higher SBNR. However, using the longer time gate will in practice result in the recording of more background counts resulting in additional noise, for instance at elevated temperatures during *operando* measurements. Therefore, the time-gated Raman spectra shown in the coming sections are all recorded with the 0.4 ns time gate.

### Comparison between time-gated and time-integrated Raman spectroscopy

**Fig. 3:**
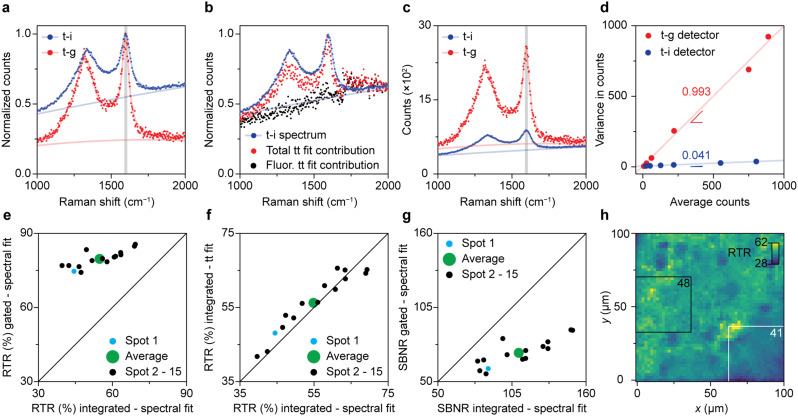
Direct comparison between time-gated and time-integrated Raman spectroscopy on a spent 0.5 wt% Pt 1.5 wt% Sn/Al_2_O_3_ propane dehydrogenation (PDH) catalyst material. (a) Comparison of the normalized time-integrated (blue) and time-gated (red) spectrum of spot 1. (b) Time-trace fit results compared with the time-integrated spectrum (blue). Time-integrated contributions of the background fluorescence (black), and total (Raman + fluorescence; red) contributions as a function of Raman shift, obtained by linear-combination fitting of the time dynamics of panel 2a, analogous to panel 2e. (c) Comparison of the time-integrated and time-gated spectrum of spot 1. The time-gated and time-integrated spectra in panels a–c are shown as datapoints together with the results of a spectral fitting procedure (Fig. S4†). (d) Noise characterization of the time-gated and time-integrated detectors. Variance against average counts for the time-gated CMOS-SPAD detector (red) and the CCD detector (blue). (e) Comparison of the time-gated and time-integrated RTR for 15 spots recorded with CMOS-SPAD array and the CCD camera, respectively. The Raman and total intensities are distinguished based on the fits as presented in panel a. (f) Comparison of the RTR based on the time-trace fits of the time-gated spectra (as in panel 2d) with the RTR based on the spectral fit of the time-integrated spectra measured on the CCD camera. (g) Comparison of the time-gated SBNR measured on the CMOS-SPAD array with the time-integrated SBNR measured on the CCD camera, for 15 spots. The noise calculations rely on the noise characterization results of panel d, and the distinction between Raman and background is based on spectral fits as in Fig. 3a. (h) Map of the RTR over an area of 100 × 100 μm^2^ of the sample surface, obtained with confocal Raman microscopy.

Fig. 3 illustrates the direct comparison between time-integrated and optimally time-gated Raman spectroscopy for the analysis of spent Pt–Sn-based PDH catalyst material. After recording a time-gated Raman dataset, the optical patch cable was switched from the time-gated spectrometer (CMOS-SPAD array) to the time-integrating spectrometer (CCD camera) and a spectrum was recorded with 4/396 of the acquisition time used in the time-resolved experiment. By doing so, the time-integrated spectrum was effectively recorded with the same acquisition time as 4 time-gated spectra of the time-resolved experiment. Both types of spectra were recorded with the pulsed excitation source of the time-gated spectrometer at the same power and focused at the same location of the sample. By doing so, any effect of pulsed-excitation-induced fluorescence saturation is the same in the two experiments.^38^

Fig. 3a–c show a visual comparison of time-gated and time-integrated spectra of the spent and coked catalyst materials. The time-gated spectrum measured on the CMOS-SPAD array and time-integrated spectrum measured on the CCD camera both show the D- and G-bands, due to coke, together with a broad background due to fluorescence (Fig. 3a). The relative intensity of the background is clearly lower with time gating. More precisely, the RTR increases from 45% to 75%, a clear improvement in spectral quality due to the rejection of a considerable amount of the background fluorescence. Fig. 3b shows that time integrating the time-trace model of Fig. 2 reproduces the spectral shape of the time-integrated spectrum recorded on the CCD camera. This confirms that the time-trace model is suitable to predict the Raman and fluorescence contributions in a time-integrated spectrum recorded on the same spot. Fig. 3c shows that the time-integrated spectrum appears less noisy than the time-gated spectrum. Indeed, the SBNR decreases from 87 to 60 when using the CMOS-SPAD array, making a comparison based on this parameter problematic.

The two spectrometers used exhibit different noise characteristics, which we have to take into account for a fair comparison of the methods. We characterize the photon counting noise in Fig. 3d and S5.† Photon counting for the time-gated detector follows Poisson statistics, with a variance roughly equal the number of counts. The variance observed with the CCD detector is also proportional to the number of counts but smaller by a factor 24. This implies some averaging procedure in the hardware or software, which we were not able to clarify with the manufacturer. Nevertheless, we can use these noise characteristics in our further comparison. They explain why in Fig. 3c the SBNR spectral quality of the time-integrated spectrum of spot 1 is 87, which is better than the SBNR of the time-gated spectrum of 60.

We found large spot-to-spot differences in the outcome of the descriptor parameters and assess the sample heterogeneity in Fig. 3e–g. For 15 spots on the sample surface, we first determine the Raman signal amplitude and background level using spectral fits (as in Fig. 3a) or using a time-trace fit (as in Fig. 2d). Fig. 3e compares the RTR measured on the time-integrating CCD camera with the RTR measured with optimal time gating on the CMOS-SPAD array. On average, the RTR increases from 55% to 80% when the optimal time gate is applied. The integrated RTR values calculated from the time trace fit are strongly correlated with the RTR values measured with the time-integrating CCD camera (Fig. 3f). This confirms the robustness of the time-trace model. The average SBNR decreases from 110 to 71 upon time gating, which is due to the better noise characteristics of our CCD camera compared to the CMOS-SPAD array. As our two detectors have such different noise characteristics (Fig. 3d), the effect of time gating can be best assessed in terms of RTR.

The spot-to-spot variations in the results of Fig. 3e–g shows that the effect of gating is location dependent. This stresses the importance of measuring on the exact same spot when comparing two detection techniques. We further studied the heterogeneous nature of the sample with confocal Raman microscopy, as shown in Fig. 3h. The map shows the RTR values at the frequency range corresponding to the G-band of 2601 spectra recorded over an area of 100 × 100 μm^2^ on a single spent and coked catalyst grain measured with a diffraction-limited laser spot. The previous experiments were performed with another microscope with a laser spot size of 41 μm in diameter. We computed the weighted mean RTR values of all square 37.6 × 37.6 μm^2^ boxes within the map to roughly mimic the averaging effect of the larger laser spot. The white and black rectangles mark the areas with the lowest, 41, and the highest, 48, weighted mean RTR.

The sample studied with time-gated Raman spectroscopy underwent some laser-induced degradation, but the effects on the spectral quality are negligible for the calculations presented above. We recorded reflection images of the sample before and after the measurement and observed that the irradiated spot became lighter. All spots exhibited some bleaching, indicated by an average grey-scale increase of 5.8% at the center of the laser spot (Fig. S6†). The effect of photobleaching on spectral quality as calculated above was assessed in a degradation experiment over 5 min and found to be negligible (Fig. S7†).

### Simultaneous *operando* time-gated and time-integrated Raman spectroscopy

The optimal time gating settings were applied for simultaneous *operando* time-gated and time-integrated Raman spectroscopy on a PDH catalyst in action. A fiber-coupled probe was used to excite the surface of the solid catalyst with a pulsed laser during the reaction. The signal was split with an optical patch cable and guided to the time-gated and time-integrated detectors simultaneously. Furthermore, the formed products were continuously analyzed with online GC.

**Fig. 4:**
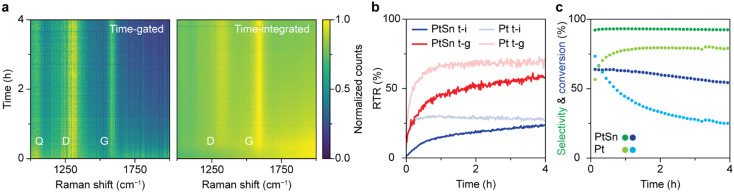
Simultaneous *operando* time-gated and time-integrated Raman spectroscopy during propane dehydrogenation (PDH) over 0.5 wt% Pt 1.5 wt% Sn/Al_2_O_3_ and 0.5 wt% Pt/Al_2_O_3_ catalysts. (a) Heatmaps of spectra simultaneously collected with time-gated and time-integrated Raman spectroscopy over the course of 4 h of PDH over the Pt–Sn-based catalyst. At each time, the spectrum is normalized to its maximum value. (b) RTR as a function of time for the time-integrated and time-gated techniques for the catalytic tests with the Pt–Sn- and Pt-based PDH catalysts. (c) Online GC results of the catalytic tests with the Pt–Sn- and Pt-based PDH catalysts.

Comparing the *operando* time-gated and time-integrated spectra over the course of 4 h of reaction (Fig. 4a) shows that time gating suppresses the background fluorescence and makes Raman bands better distinguishable, especially in the beginning of the reaction. The spectra were normalized to their maximum value to visualize their most pronounced features. The D (1320 cm^−1^) and G (1600 cm^−1^) bands, associated with coke deposits, are clearly visible in both heatmaps. In addition, a band at 1050 cm^−1^ is observed that can be ascribed to Si–O vibrations of the quartz reactor.^5^ The time-gated data show some broadband background fluorescence between 1800 and 2000 cm^−1^ at the beginning of the experiment, which disappears within 30 min. The initial intense fluorescence is more pronounced in the time-integrated data. Here, the D and G band are barely distinguishable above the background fluorescence in the first 30 min of the experiment. The initial fluorescence is likely caused by polycyclic aromatic molecules that are the precursors of coke deposits. In the initial stages of the reaction, aromatics are formed by dimerization of propyl species due to side reactions, such as cracking and deep dehydrogenation.^43,44^ In later stages, the aromatics grow into graphitic sheets covering the catalyst and losing their fluorescence.^43,44^ Indeed, the heatmaps of non-normalized spectra reveal that the absolute fluorescence intensity peaks after 2 min after which it decreases with time reaching half its intensity after 48 min. For the Pt-based catalyst the fluorescence decreases instantly and halves after 2 min (Fig. S8†). The coke bands appear immediately for the Pt-based catalyst, while these bands appear gradually for the Pt–Sn based catalyst, reaching a maximum after 55 min (Fig. S8†). These trends reveal that the Pt-based catalyst seems to undergo quicker coking compared to the Pt–Sn-based catalyst. After reaching their maximum, the absolute intensities of the Raman signals (D-band, G-band, and quartz band) slowly decrease. This is likely the result of coking, which turns the sample black over the course of hours and decreases light penetration.

Fig. 4b quantifies the interpretability of the spectra over time in terms of RTR for the Pt–Sn- and the Pt-based catalyst. The RTR of the spectra recorded with time gating is higher than for the time-integrated spectra, especially in the beginning of the reaction. These results highlight that measuring in a time-gated fashion enables better distinction of the Raman features, especially in the early coking stages where spectra are dominated by fluorescence.

The online GC data, shown in Fig. 4c, provide more insight in the catalyst deactivation processes. For the Pt–Sn-based catalyst, the selectivity towards propylene is 90% and reaches its maximum after 12 min after which it stays constant. In the first 12 min, the products of side (cracking) reactions, methane, ethane, and ethylene, are formed in considerable quantities. This can indicate that first the sites active for side reactions are blocked by fast forming coke deposits. The gradual decrease in propane conversion together with the gradual appearance of the Raman bands after 30 min of PDH reaction can be attributed to the blockage of the active sites for the dehydrogenation reaction due to coking. The online GC data shows that the Pt-based catalyst material behaves differently compared to the Pt–Sn-based catalyst. The propylene selectivity for this catalyst is considerably lower. Hence, the Pt-based catalyst shows more activity towards products of side (cracking) reactions that result in coking of the catalyst. Furthermore, the conversion of propane decreases quicker, indicating faster blockage of active sites, which is also indicative of faster coking behavior, analogous to the immediate appearance of the Raman bands due to coke. Combining online GC and Raman data shows that the Pt-based catalyst cokes faster compared to the Pt–Sn-based catalyst. This difference is due to the absence of Sn, a well-known promoter that plays a key role in the stabilization of the catalyst and suppression of coke formation by modifying the electronic properties of Pt.^43,44^

## Experimental

### Materials

The following solid catalysts and other materials have been used in this study: 0.5 wt% Pt/Al_2_O_3_ (BASF, 150–425 μm sieve fraction, surface area of 85.2 m^2^ g^−1^; and pore volume of 0.59 cm^3^ g^−1^), 0.5 wt% Pt 1.5 wt% Sn/Al_2_O_3_ (BASF, 150–425 μm sieve fraction; surface area of 79.6 m^2^ g^−1^; and pore volume of 0.56 cm^3^ g^−1^), Pt(NO_3_)_2_·*n*H_2_O (Heraeus, 57.76 wt% Pt; impurities Ir + Pd + Rh + Ru ≤ 500 ppm, *P* ≤ 100 ppm, *S* ≤ 100 ppm), Sn(ii)Cl_2_·2H_2_O (Sigma Aldrich, 62.26 wt% Sn, >99.99% pure), Rhodamine 6G (Acros Organics, 99% pure) and γ-Al_2_O_3_ (Alfa Aesar, surface area of 240.2 m^2^ g^−1^; and pore volume of 0.82 cm^3^ g^−1^).

### Catalyst preparation

The 0.5 wt% Pt/Al_2_O_3_ catalyst was prepared by impregnating an aqueous Pt(NO_3_)_2_ solution onto the Al_2_O_3_ support. The impregnated catalyst was subsequently dried at 120 °C for 30 min and calcined in air at 560 °C for 3 h. The 0.5 wt% Pt 1.5 wt% Sn/Al_2_O_3_ was prepared by impregnating an aqueous Pt(NO_3_)_2_ solution onto the Al_2_O_3_ support. The impregnated catalyst was subsequently dried at 120 °C for 30 min. In a second impregnation step the Sn(ii)Cl_2_ was impregnated onto the catalyst, dried at 120 °C for 30 min and calcined in air at 560 °C for 3 h.

### Propane dehydrogenation

Propane dehydrogenation (PDH) experiments were performed in a lab-scale reactor with a method adapted from Sattler *et al.* (Fig. S1†).^5,37^ 300 mg of the catalyst material was placed in a packed-bed-type quartz reactor. The catalyst bed was sealed with a layer of quartz wool. The catalyst was first heated from room temperature to 600 °C at a rate of 10 °C min^−1^ under a H_2_ flow (8 mL min^−1^) to reduce the catalyst to its catalytically active metallic state. The reactor was then purged with He (8 mL min^−1^) for 5 min. The dehydrogenation started when propane was introduced to the reactor at a flow rate of 8 mL min^−1^. At the same time, the online gas chromatography (GC) program as well as the simultaneous time-resolved time-integrated and time-gated Raman spectroscopy experiments (see below) were initiated. A gas chromatogram (GC) was recorded every 6 min with a Compact GC 4.0 (Global Analyser Solutions) equipped with two Rt-UBond columns (2 m × 0.32 mm and 8 m × 0.32 mm) and a flame ionization detector (FID). After 4 h of reaction, the gas feed was switched to He (8 mL min^−1^) and the oven was cooled down to room temperature. The samples were collected for *ex situ* analysis.

### Time-gated and time-integrated Raman spectroscopy

The Raman spectroscopy experiments presented in this work were carried out with two types of spectrometers. The time-integrated spectra were recorded with the AvaRaman-532 HERO-EVO (Avantes) spectrometer equipped with a 25 μm slit, a HSC1200-0.75 grating, and a back-thinned TE cooled CCD Detector with 1024 × 58 pixels. The time-gated spectra were recorded with the PicoRaman M1 (Timegate Instruments) equipped with 768 × 8 SPAD pixels. The pulsed 532 nm laser (150 kHz, 150 ps FWHM) of the PicoRaman was used as the excitation source. We refer to the devices as time-integrated and time-gated spectrometers.

In the case of time-gated Raman spectroscopy, a pulsed laser is used to excite the catalyst sample, which typically induces both Raman scattering and fluorescence. The resulting signal is guided towards the time-gated spectrometer with optical patch cables. After entering the time-gated spectrometer, the signal pulse falls on a dispersion element. Light with wavelengths between 515 and 600 nm falls on the detector composed of 8 × 768 SPAD pixels (6 sub detectors with 8 × 128 pixels each). The time gating occurs at the detector. The SPAD pixels are switched on after a delay time with respect to the laser pulse. By doing so, a fraction of the signal pulse is recorded. All 768 pixels in the wavelength dimension are equipped with 8 sub pixels, constituting 4 pairs that are sequentially activated for 100, 200, 100, and 1000 ps, respectively. By combining the signal of the different detector pairs, one has access to time gates of 100, 200, 300, 400, 1000, 1100, 1300, and 1400 ps. A time-gated Raman spectrum is obtained by counting the photons that arrive within the time bins after the set delay time, for a set number of laser pulses. This process is repeated for a range of delay times yielding a time-resolved Raman spectrum, *i.e.*, the recorded spectrum as a function of delay time after the laser pulse. The detector can be activated up to about 6 ns after the arrival of the laser pulse. The software scans through the range of delay times multiple times to avoid artefacts due to sample degradation occurring on the time scale of minutes. We report the number of laser pulses used to probe the Raman spectrum at each delay time.

### Time-gated and time-integrated Raman microscopy

A spent Pt–Sn-based PDH catalyst material was analyzed with time-gated and time-integrated Raman microscopy (Fig. S1†). An upright Olympus microscope (Olympus BX41M) mounted with the Raman Microprobe (Timegate Instruments) was coupled to both spectrometers with optical patch cables to allow for time-gated and time-integrated Raman measurement at the same spot *via* the same optical path. A 50× 0.8 NA Olympus objective with a working distance of 0.66 mm was used for the measurements. The pulsed laser of the PicoRaman was used as the excitation source, delivering a power of 6.2 mW (measured with an Ophir Orion PD power meter) and illuminating an area of 41 μm in diameter.

The microscopy set-up was used in a procedure developed to study the temporal shape of the Raman signal and directly compare time-gated and time-integrated Raman spectroscopy. The sample under study was the coked 0.5 wt% Pt 1.5 wt% Sn/Al_2_O_3_ PDH catalyst. First, a time-gated Raman dataset was recorded with the full range of delay times to obtain as much as possible of the temporal shape of the signal pulse. The time-gated spectra were recorded for delay times between 4.30 and 10.21 ns in 396 steps of 15 ps. At each delay time, the spectrum was integrated for 70 000 laser pulses at a repetition rate of 150 kHz resulting in a total acquisition time of 185 s. Subsequently, the collection path was switched to the time-integrated spectrometer to record a time-integrated spectrum, while using the same excitation source and power. Here, we used an integration time of 1.87 s, which is shorter than the total acquisition time of the time-resolved Raman spectrum by a factor 396/4. By doing so, the acquisition time of the time-integrated spectrum is equal to that of the sum of time-gated spectra of four delay times. The time-integrated spectrum and the sum of four time-gated spectra can be directly compared as the acquisition time, location on the sample, laser spot size, and laser power used were equal. The recording of the spectra is preceded by the acquisition of a spectrum with the same settings in the absence of the excitation source. This measurement is denoted as the dark spectrum. Reflection images of the spot before and after laser exposure were recorded with a CS165CU/M 1.6 MP Color CMOS Camera (Zelux) that was integrated in the Microprobe. The images were used to assess laser-induced sample degradation. The procedure was followed to analyze 15 spots.

### Simultaneous *operando* time-gated and time-integrated Raman spectroscopy

The formation of carbon deposits on PDH catalyst material was studied with simultaneous *operando* time-gated and time-integrated Raman spectroscopy. We subjected the Pt- and Pt–Sn-based catalyst materials to the PDH procedure described above. The quartz reactor was equipped with a rectangular window that was aligned with a hole in the oven that allowed for the study of the catalyst bed with a fiber-coupled optical probe. An optical patch cable was used to deliver the light from the time-gated spectrometer to the probe (Thorlabs multi-mode, 1 m length, 0.22 NA, 105 μm core diameter). The probe was composed of a HFPH-FC-S-532 filter box (Kaiser Optical Systems Inc.) and an in-house designed head based on a 9.525 mm diameter sapphire ball lens (Edmund Optics Inc.) that focused the laser light on the sample at 0.71 mm and collected the resulting signal. The laser power at the probe head was 25 mW, measured with an Ophir Orion PD power meter. The collected signal passed through the head, the filter box, and an optical patch cable (Thorlabs, multi-mode, 1 m length, 0.22 NA, 200 μm core diameter) and was split with a 1 × 2 optical patch coupler (Thorlabs, multi-mode, 0.22 NA 200 μm core diameter, 50/50 coupling ratio, 15 cm custom lead lengths) to deliver the signal to the time-gated and time-integrated spectrometer simultaneously.

The catalyst was activated in H_2_ and heated up to 600 °C, after which it resided in a He flow. During this step, both types of Raman spectra were recorded in the absence of laser light to be used for the background correction. Hereafter, propane was introduced to the system for 4 h. At the same time, the online GC program as well as the simultaneous time-resolved time-integrated and time-gated Raman experiments were initiated. Half of the signal was delivered to the time-integrated spectrometer where a spectrum was recorded every minute (5 s integration time, averaged 12 times). The other half to the signal was recorded with time-gated spectrometer. The time-gated spectra were recorded for 5 delay times; 5.37, 5.38, 5.39, 5.40, and 5.41 ns. At each delay time, the spectrum was integrated for 884 975 laser pulses at a repetition rate of 150 kHz resulting in a total experiment time of ∼30 s. Effectively, however, we recorded one spectrum every minute when using these settings, due to software related delay during the initiation of a measurement and the processing of the results.

### Spectral data processing

The time-gated data was first processed in the SHSGUI software (Timegate Instruments) to compensate for non-uniformity in pixel response of the SPAD array. The ‘delay correction’ corrects for width and offset variations in the time bins. Convolution compensates for both while translation only compensates for the offset variation. In the literature, pixel-to-pixel timing heterogeneity, so called timing skew, is estimated to cause more distortion in the time-gated spectra than Poisson noise.^38–40^ A close inspection showed that, after delay correction, the time-gated Raman spectra exhibited Poisson noise (Fig. S2†). Furthermore, a saturation correction was performed to compensate for the arrival of multiple photons at the SPAD that can only register one count per activation. The delay times were shifted so that the maximum of the laser peak is positioned at 0 ns.

Both the time-gated and time-integrated spectra were processed further by subtracting the background spectrum. The resulting spectra were corrected for the transmission of the optics and the pixel response of both detectors used in the *operando* and microscopy experiments. The recorded wavelengths (nm) were converted to Raman shift (cm^−1^) using the laser wavelength of 532.21 nm (Fig. S2†).

### Confocal Raman microscopy

A confocal Raman microscope (Horiba Scientific) was used to acquire spatial information on the nature of the carbon deposits on the surface of the spent Pt–Sn-based catalyst material. A 50× 0.5 NA objective with a working distance of 10.6 mm was used to focus a 532 nm laser that delivered 0.52 mW to the sample (measured with an Ophir Orion PD power meter) illuminating a diffraction-limited spot. A long pass edge filter in the detection path was used to block the laser light and a 1200 lines per mm grating was used as dispersion element.

### Noise characterization

The photon counting noise of both spectrometers was characterized with a method adopted from van Swieten *et al.*^41^ The light of a deuterium/halogen lamp (AvaLight-DH-S-BAL) was led towards the spectrometers under study *via* optical patch cables and a cube that facilitated the placement of several neutral-density filters in the optical path. Subsequently, we have recorded a large number of spectra for several illumination intensities. The time-integrated spectrometer was used to record 2500 spectra with 100 ms integration time. The time-gated spectrometer was used to record 250 spectra. The time-gated spectra were recorded for 5 delay times; 4.66, 4.67, 4.68, 4.69 and 4.70 ns. At each delay time, the spectrum was integrated for 350 000 laser pulses at a repetition rate of 150 kHz.

## Conclusions

We have explored, optimized and validated time-gated Raman spectroscopy based on the signal dynamics of the G-band due to graphitic carbon deposits on spent propane dehydrogenation catalyst materials. We were able to distinguish the G-band Raman scattering from background fluorescence based on their different time dynamics. A time-trace model helped us determine the time gating that yields the optimal signal to background noise ratio. A fast, sub ns component of the fluorescence made complete suppression of background fluorescence by time gating difficult.

Optimally time-gated Raman spectra of a spent propane dehydrogenation catalyst showed clearly lower background fluorescence compared to the time-integrated Raman spectra. As the two detectors exhibited different noise characteristics, the effect of time gating could be best assessed in terms of the Raman-to-total ratio, instead of the signal-to-background-noise ratio. The spot-to-spot variations showed that the effect of time gating is location-dependent and stresses the importance of measuring on the exact same spot when comparing the two detection techniques.

The simultaneous *operando* time-gated and time-integrated Raman spectroscopy experiments demonstrated the benefit of time gating. The Raman-to-total ratio of the spectra recorded with time gating is higher than the time-integrated spectra, especially in the beginning of the propane dehydrogenation reaction over Pt/Al_2_O_3_ and Pt–Sn/Al_2_O_3_ catalysts. These results highlight that measuring in a time-gated fashion enables better distinction of the Raman features, especially in the early coking stages of solid catalysts where Raman spectra were dominated by background fluorescence.

To illustrate the versatility of the analytical approach proposed, we have explored the potential of time-gated Raman spectroscopy for zeolites. Classical Raman spectroscopy of zeolite-based materials is known to be complicated by a broad and intense background fluorescence.^45,46^ The results obtained are shown in Fig. S9.† These preliminary data will form the basis for future time-gated Raman spectroscopy studies in our research group.

## Author contributions

B. M. W. conceived the initial ideas of the PhD project, wrote the project, and acquired the related funding. B. M. W. and F. T. R. designed and supervised the research. R. V. performed the experimental work and analyzed the data. R. V., P. T. P. and F. T. R. constructed the time-trace model. R. V., P. T. P., F. T. R. and B. M. W. contributed to the interpretation of the results. R. V. wrote the manuscript with feedback from all authors.

## Conflicts of interest

The authors declare no conflict of interest.

## Supplementary Material

CY-013-D3CY00967J-s001
