# Supplementary material for: Operando time-gated Raman spectroscopy of solid catalysts
Source: Catal Sci Technol. 2023 Sep 7;13(22):6366–76. doi: 10.1039/d3cy00967j (PMC10642357; doi:10.1039/d3cy00967j)
Supplement: CY-013-D3CY00967J-s001 [file CY-013-D3CY00967J-s001.pdf]

This journal is © The Royal Society of Chemistry 2023

1. Details of Experimental Setups

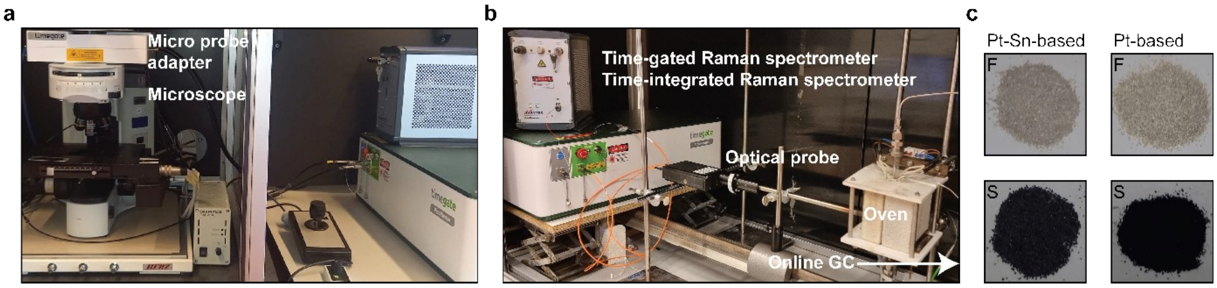

Figure S1. Experimental details of the setup and materials used. (a) Setup used for time-gated and time-integrated Raman microscopy. (b) Setup used for simultaneous *operando* time-gated and time-integrated Raman spectroscopy. (c) Pt-Sn-based PDH catalyst material, 0.5 wt% Pt 1.5 wt% Sn/Al_2O_3, before (F) and after 4 h PDH (S) and Pt-based PDH catalyst material, 0.5 wt% Pt/Al_2O_3, before (F) and after 4 h PDH (S).

2. Spectral Processing

Figure S2 shows how the time-gated and time-integrated spectra are corrected for the transmission of the optics of the microscopy setup and the pixel response for both detectors. It is common to use the known spectrum of a calibration lamp for correction purposes. It was, however, not possible to record a reference lamp spectrum with the time-gated spectrometer with sufficient counts. The SPAD detector used can only be switched on for a period of 1.4 ns per laser pulse. Working with 150 kHz laser pulse effectively allows to collect photons in a very small fraction of the measurement time. Therefore, pulsed laser induced fluorescence from a γ-Al_2O_3 sample impregnated with a Rhodamine 6G (Rh6G) dye solution was used for reference purposes.

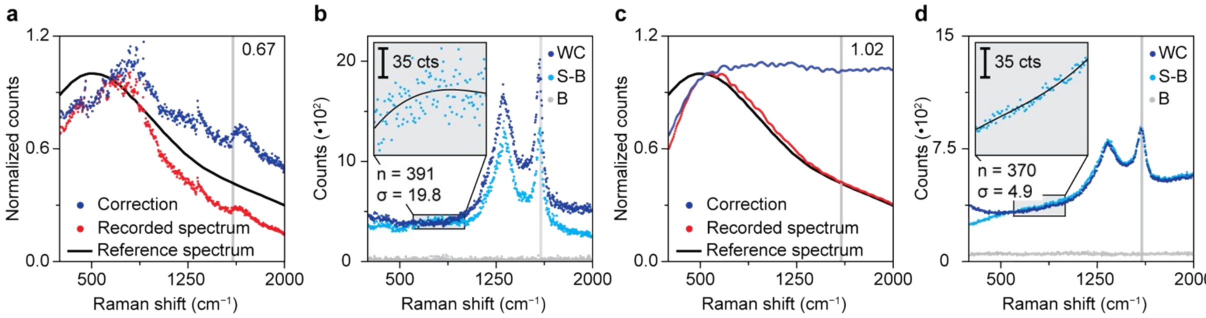

Figure S1. Data processing for time-gated and time-integrated Raman spectroscopy. (a) Reference Rh6G and time-gated Rh6G microscopy spectra and the resulting correction curves measured on the CMOS-SPAD array. (b) Processing of the delay-corrected time-gated Raman spectrum of a spent PDH catalyst material. The spectrum recorded in the absence of laser light (D) is subtracted from delay corrected spectrum (S-D), which is divided by the correction curve to obtain the corrected spectrum (WC). The inset shows a polynomial background fit. (c) Reference Rh6G and time-integrated Rh6G microscopy spectra and the resulting correction curves measured on the CCD camera. (d) Processing of the raw time-integrated Raman spectrum of a spent PDH catalyst material. The spectrum recorded in the absence of laser light (D) is subtracted from the raw spectrum (S-D), which is divided by the correction curve to yield the corrected spectrum (WC).

Figure S2a illustrates how a sensitivity correction curve for the microscopy setup and the time-gated Raman spectrometer is constructed. A reference Rh6G spectrum, recorded with a calibrated spectrometer is presented together with a Rh6G spectrum recorded with the microscopy setup and the time-gated Raman spectrometer. The time-gated spectra were recorded for delay times between 4.2 and 7.0 ns in 94 steps of 0.3 ns. At each delay time, the spectrum was integrated for 1,750,000 laser pulses at a repetition rate of 150 kHz. The spectrum was delay-corrected in the SHSGUI software and background-corrected with a spectrum recorded in the absence of the excitation source. The recorded spectrum was divided by the reference spectrum, resulting in a correction curve.

Figure S2b shows how the time-gated Raman spectrum of spent Pt-Sn-based PDH catalyst material is corrected for the transmission of the optics of the microscopy setup and the pixel response of the detector. The delay- and background-corrected spectrum is divided by the correction curve. Effectively this operation increases the counts with a factor of 1.5 (1/0.67) in the G-band region between 1590 and 1610 cm^{-1}. This correction factor (CF) plays a role in the signal-to-background-noise ratio (SBNR) calculations. The inset shows that the background signal contains Poissonian noise: the background-corrected standard deviation (BGSD) is roughly equal to the square root of the average counts.

Figure S2c illustrates how a sensitivity correction curve for the microscopy setup and the time-integrated Raman spectrometer is constructed. The reference Rh6G spectrum is shown together with a background-corrected Rh6G spectrum recorded with the time-integrated spectrometer. The latter spectrum is recorded with 60 s integration time. The recorded spectrum was divided by the reference

spectrum, resulting in a correction curve. The wave-like features are attributed to the transmission of the microscope optics.

Figure S2c shows how the time-integrated Raman spectrum of spent Pt-Sn-based PDH catalyst material is corrected for the transmission of the optics of the microscopy setup and the pixel response of the detector. Here, the operation does not affect the counts in the G-band to a large extent, so we used CF = 1 in our estimation of the noise (eq 1 of the main text). The inset illustrates a considerably smaller deviation in background counts compared to the time-gated spectra. The BGSD is much smaller than expected from Poisson statistics. Figure S5 describes the noise characteristics of both spectrometers are in more detail.

The correction curves for the spectra recorded with the high-temperature probe are constructed in a similar fashion. The Rh6G powder is placed in the quartz reactor and the spectra are recorded with the fiber coupled optical probe to mimic the conditions of the catalysis samples.

3. Signal Shape as Observed with the Time-Gated Raman Spectrometer

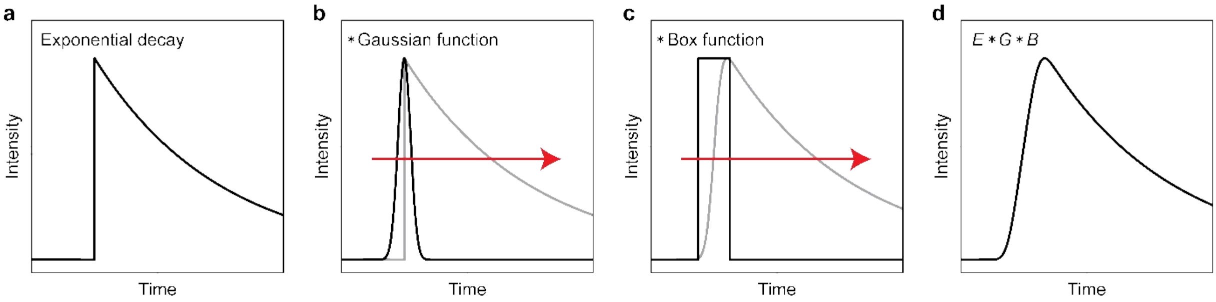

Figure S3. Convolution of exponential decay with the Gaussian IRF and a box function. The convolution yields the temporal shape of fluorescence, denoted with E*G*B, as recorded with the CMOS-SPAD-based time-gated Raman spectrometer.

Figure S3 shows how temporal shape of fluorescence as observed with the time-gated Raman spectrometer is built up. In general, fluorescence decays exponentially following pulsed laser excitation. This shape is convoluted with a Gaussian function for the IRF and convoluted with a box function with the width of the active time of the pixel, $E * G * B$.

The function E is used for exponential decay:

$$E(t) = \begin{cases} 0 & ;\ t < t_0 \\ \frac{1}{\tau} e^{-\frac{t - t_0}{\tau}} & ;\ t > t_0 \end{cases} \qquad \text{Eq. S1}$$

where τ is the fluorescence lifetime, and t_0 the starting point of the emission. The normalized IRF is described with a Gaussian function $G(t)$:

$$G(t) = \frac{1}{\sqrt{2\pi}\,\sigma} e^{-\frac{1}{2}\left(\frac{t - t_0}{\sigma}\right)^2} \qquad \text{Eq. S2}$$

where σ is the standard deviation. The convolution between E and G yields the following function:

$$(E * G)(t) = \frac{e^{\frac{\sigma^2 - 2(t - t_0)\tau}{2\tau^2}}\left(1 + erf\left[\frac{-\sigma^2 + (t - t_0)\tau}{\sqrt{2}\sigma\tau}\right]\right)}{2\tau} \qquad \text{Eq. S3}$$

The time-gated Raman spectrometer probes the temporal shape of the signal ($E * G$), by switching on the SPAD for discrete times with respect to the laser pulse. This binning can be described with a box function, $B(t)$:

$$B = \begin{cases} 1 & ;\ |t| \le \frac{1}{2}\delta \\ 0 & ;\ |t| > \frac{1}{2}\delta \end{cases} \qquad \text{Eq. S4}$$

where δ is the width of the time bin. The convolution of B with the signal shape $E * G$ yields:

$$(E * G * B)(t) = \frac{1}{2}\left\{ -1 + erf\left[\frac{2(t-t_0)+\delta}{2\sqrt{2}\sigma}\right] + erfc\left[\frac{2(t-t_0)-\delta}{2\sqrt{2}\sigma}\right] + e^{\frac{\sigma^2-(2(t-t_0)+\delta)\tau}{2\tau^2}}\left[-2 + e^{\frac{\delta}{\tau}} \right.\right.$$

Eq. S5

This function contains optimizable parameters t_0,δ,σ, and τ.

$$(E * G * B)(t) = f(t_0,\delta,\sigma,\tau)$$

Eq. S5

The time trace can fitted with a sum of multiple $f(t_0,\delta,\sigma,\tau)$ components with a contribution factor h_i. The linear combination of multiple components is described with a sum $ExGxB_i$.

$$(E * G * B)(t)_i = \sum_i f(t_0,\delta,\sigma,\tau_i,h_i)$$

Eq. S6

The time trace of the Raman band and fluorescence signal are best fitted with 2 and 3 components respectively. The G-Band time trace, composed of both Raman and Fluorescence is fitted with a linear combination of the Raman fit and the fluorescence fit.

4. Spectral Fitting Procedure

The recorded spectra were composed of a broad background with several Raman features. It is necessary to distinguish between background and Raman signal in order to assess spectral quality descriptors such as the RTR and the SBNR. The separation can be accomplished with a spectral fit. In this work we fitted the spectra data to a linear combination of Lorentzian line shapes with p_i for the position w_i for the width and h_i for the area of the band and a 3rd degree polynomial function for the background.

$$f(x) = ax^2 + bx + c + \sum_i \frac{h_i}{\pi} \frac{0.5\, w_i}{(x - p_i)^2 + (0.5\, w_i)^2}$$

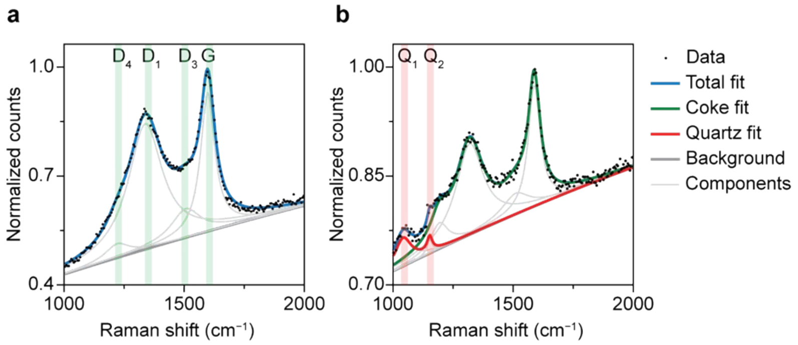

Figure S4. Results of the fit of the Raman spectra of (a) a coked catalyst sample and (b) a coked catalyst sample in a quartz reactor (b). The contributions of the bands that make up the spectrum are plotted as individual components in light grey. The contribution of the quartz and coke bands in (b) are shown in red and green respectively.

Figure S4 shows the results of the spectral fitting procedure. We encountered only two distinctly different Raman spectra in this work. The first spectrum shown in Figure S4a is typical for coked catalyst material under the microscope. The second spectrum, in Figure S4b, is observed when the coked catalyst resides in the quartz reactor. The spectra can be fit to a function with 4 and 6 Lorentzian line shapes respectively. The G and D_1 (denoted D in the main text) are the dominant features due to ring vibrations of an ideal graphitic lattice and ring vibrations of a disordered graphitic lattice respectively. Other minor features, D_3 and D_4 are due to amorphous carbon and a disordered graphitic lattice. The spectrum of the sample in the quartz reactor exhibits two additional bands, Q_1 and Q_2 due to the Si-O stretch vibrations of quartz.[1]

5. Noise Characterization

The photon counting noise of both spectrometers was characterized with a method adopted from van Swieten et al. [2]. The light of a Halogen lamp (AVaLight-DH-S-BAL) was led towards the spectrometers under study via optical patch cables and a cube that facilitated the placement of several OD filters in the optical path. Subsequently, we recorded a large number of spectra for several illumination intensities.

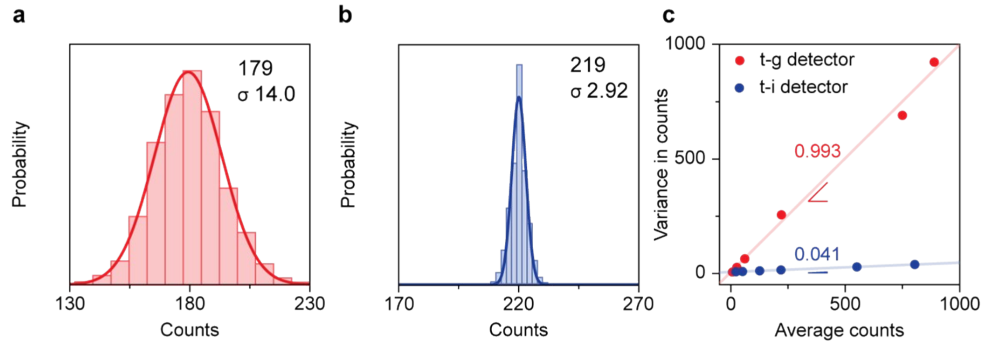

Figure S5. Noise characterization of the time-gated and time-integrated Raman spectrometers. (a) Probability distribution of the counts of pixels that showed the mode of 179 recorded with the time-gated SPAD spectrometer. (b) Probability distribution of the counts of pixels that showed the mode of 219 recorded with the time-integrated CCD spectrometer. (c) Variance against average counts for the two spectrometers.

Figure S5 describes the characterization of the photon counting noise of the time-gated and time-integrated spectrometers. A series of spectra recorded with one illumination intensity was averaged per pixel. All the counts of the pixels with the mode average were combined in order to make sure that we study pixels with the same behavior. A probability distribution of the counts for both spectrometers at a specific illumination intensity is shown in Figure S5 (a and b). The distributions are fitted with a Gaussian function to obtain the standard deviation σ. The procedure is followed for sets of spectra recorded with several transmissions, yielding several average counts. The variance, σ^2, is plotted against the average counts in Figure S5c to illustrate the noise characteristics of the two spectrometers. Photon counting for the time-gated detector follows Poisson statistics, with a variance roughly equal the number of counts. The variance observed with the CCD detector is also proportional to the number of counts but smaller by a factor 24. This implies some averaging procedure in the hardware or software, which we were not able to clarify with the manufacturer.

6. Sample Stability Test – Grey Scale Analysis

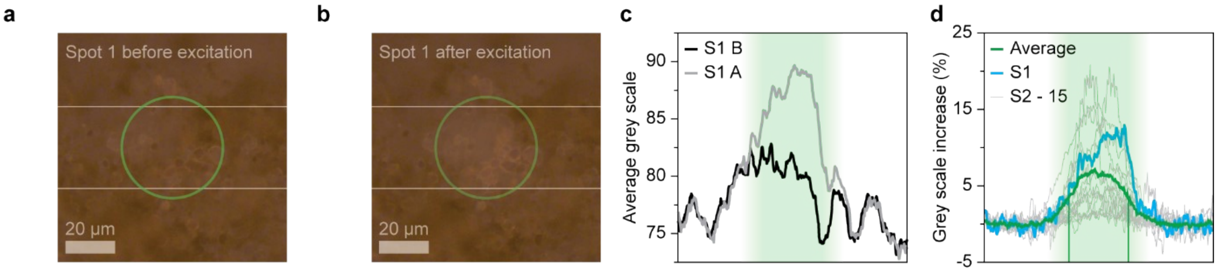

Figure S6. Grey-scale analysis of the white-light microscopy images before (a) and after (b) the recording of a time-gated and time-integrated spectra. The green circle represents the laser spot with a 41 μm diameter. (c) Average grey-scale before (B) and after (A) excitation of spot 1 of the areas marked by the horizontal grey lines in (a) and (b). (d) Grey-scale increase and average grey-scale increase for spots 1–15. The area illuminated with the laser in (c) and (d) is illustrated with the green shading. The area over which the average grey-scale increase is calculated is marked with the green vertical lines. The grey scale increase was corrected for variation in average grey scale of the unilluminated area due to fluctuations in lamp intensity.

Figure S6 illustrates how we studied laser induced sample degradation with a grey-scale analysis of the reflection images of the catalyst material before and after the Raman measurements. The white-light images recorded before and after excitation showed a slightly whiter area after excitation at the position of the laser spot, as shown in Figures S6a,b. The laser radiation causes some of the black carbon deposits to bleach, exposing more of the lighter catalyst material. This faint effect was assessed with a grey-scale analysis of the images. Figure S6c shows the grey-scale value as a function of the *x*-coordinate over a line running through the center of the laser spot and averaged over a 30-μm-wide band in the *y*-direction (horizontal lines in Figures S6a,b. The area illuminated by the laser showed a significant increase in grey-scale value, while the grey scale of the unilluminated area barely changes. This observation indicates laser-induced bleaching. The analysis was repeated for all 15 spots. The grey-scale increase as a function of the *x*-coordinate is shown in Figure S6d. All spots exhibited bleaching to some extent, indicated by an average grey-scale increase of 5.8% at the center of the spot.

7. Sample Stability Test – Spectral Change

We observed laser-induced bleaching while carrying out the ex-situ sample analysis. Sample stability tests were carried out to determine whether this could have an influence on de outcome of our calculations. The acquisition of the time-resolved Raman spectrum with the CMOS-SPAD array (Figure S2a) took about 4 min and the subsequent collection of the time-integrated spectrum with the CCD camera on the same spot took roughly 1 s. To evaluate the effect of sample degradation over the course of an experiment, we collected a series of 130 time-integrated spectra over a total time of 5 min, using the same pulsed excitation as in the experiments of the main text.

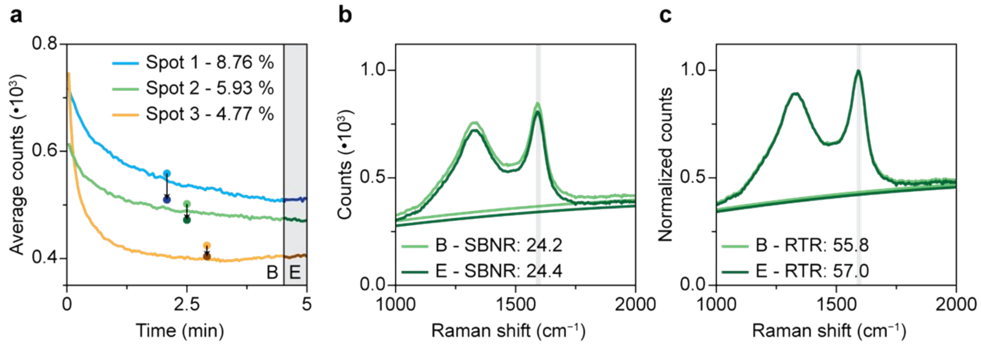

Figure S7. Effects of laser induced sample degradation on spectral quality descriptors. The sample under study is the spent 0.5 wt% Pt 1.5 wt% Sn/Al_2O_3 PDH catalyst. (a) Average counts over time. The average counts of the first 4.5 min (beginning; B) are compared with the average counts observed at the final 0.5 min (end; E). (b) Average spectra at the beginning and the end of the experiment to assess the SBNR. (c) Average normalized spectra at the beginning and the end of the experiment to assess the RTR. All shown spectra, where fitted with the sum of 4 Lorentzian line shapes and a polynomial background to discriminate the background from the Raman scattering.

Figure S7 illustrates the effect of laser induced sample degradation on the spectral quality descriptors SBNR and RTR. Figure S7a shows a fast decrease in average counts at the beginning of the stability test. This resulted in an average counts loss of 4.77 – 8.77%, when comparing the average spectrum of the beginning (B) of the experiment with the average spectrum at the end (E) of the experiment. The change in quality descriptors, SBNR (Figure S7b) an RTR (Figure S7c) however was small when comparing B to E. Fluorescence decrease due to photon bleaching resulted in small, but negligible improvements for both quality descriptors.

8. Additional Results of Simultaneous *Operando* Time-Gated and Time-Integrated Raman Spectroscopy

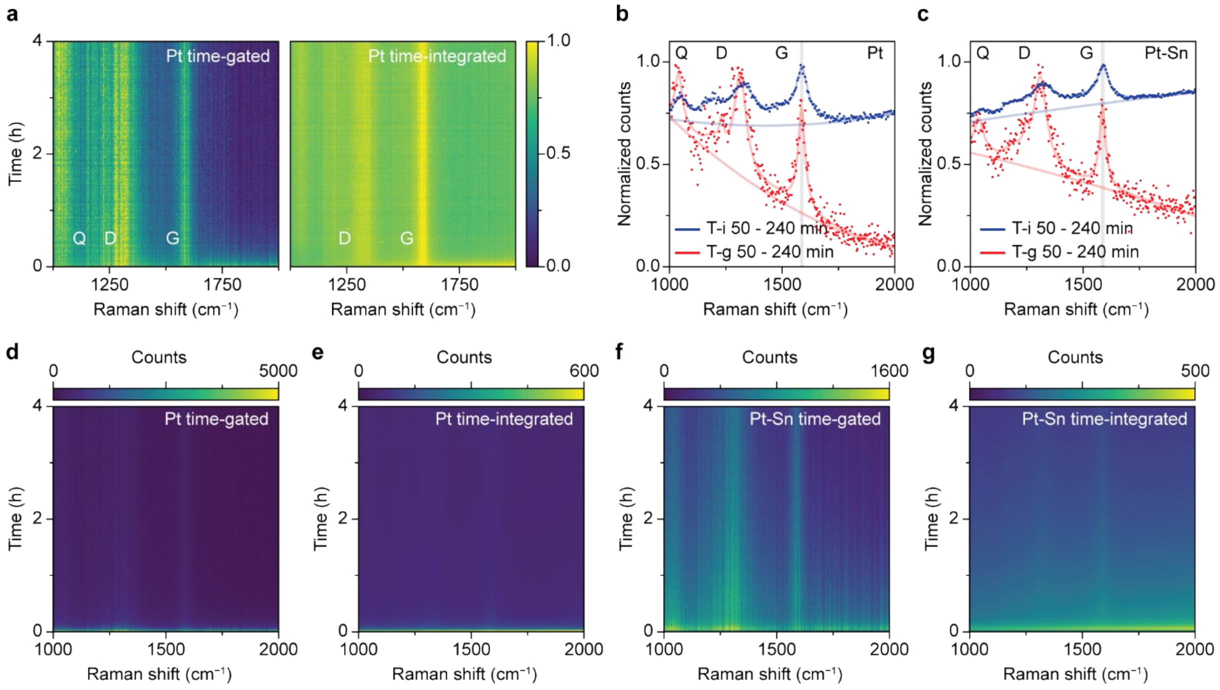

Figure S8. Simultaneous *operando* time-gated and time-integrated Raman spectroscopy during propane dehydrogenation over 0.5 wt% Pt 1.5 wt% Sn/Al_2O_3 and 0.5 wt% Pt/Al_2O_3 PDH catalysts. (a) Heatmaps with normalized spectra simultaneously collected with time-gated and time-integrated Raman spectroscopy during 4h of propane dehydrogenation over Pt-based catalyst. (b,c) The normalized average time-integrated and time-gated spectra recorded between 50 and 240 minutes for the Pt-based (b) and Pt-Sn-based catalysis (c). Heatmaps of the spectroscopy data with the spectra that were not normalized (e) (f) (g) (h).

Figure S8 shows additional results obtained during the *operando* spectroscopy experiments. Figure S8a shows the normalized time-gated and time-integrated Raman spectra of the Pt-based catalyst. The spectra are dominated by the Q, D, and G bands. The coke bands appear instantaneously, indicative for very fast coke formation. The normalized average time-gated and time-integrated spectra obtained during PDH over the Pt- and Pt-Sn-based catalysts are depicted in Figure S8b and S8c. The spectra show that the RTR increases strongly when the time gate is applied. In addition, the backgrounds of the time-integrated spectra are rather flat and intense while the backgrounds of the time-gated spectra are more tilted. The heatmaps of the *operando* experiments, composed of non-normalized spectra are

shown in Figures S8d-g. The heatmaps are dominated by intense fluorescence that disappears after the first minutes for the Pt-based catalyst. The Pt-Sn-based catalyst materials show intense fluorescence that decays slowly over time.

9. Time-Gated and Time-Integrated Raman Spectroscopy for Zeolite Materials

To show the general applicability of the proposed methodology a zeolite Y (CBV 720 Zeolyst) sample was subjected to the time-gated and time-integrated Raman microscopy method, as described in the Experimental section, with the aim to optimize time gating and directly compare the optimally time-gated Raman spectrum to its time-integrated Raman counterpart. A laser power of 7.5 mW (measured with an Ophir Orion PD power meter) was used in this experiment.

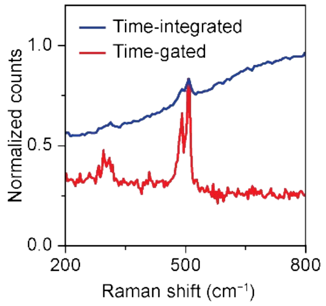

Figure S9. Direct comparison between normalized optimally time-gated (red) and time-integrated (blue) Raman spectra of zeolite Y. The spectra were normalized using the maximum intensity of the band between 475 and 525 cm^{-1}.

Figure S9 illustrates the potential of time gating for enhanced Raman spectroscopy of zeolite material. The Raman band between 475 and 525 cm^{-1} due to a characteristic vibrational mode of the zeolite framework is barely visible above the background fluorescence in the time-integrated spectrum.[3] It is clear that time gating suppresses the fluorescence background and also enhances the RTR from 12 to 65%.

References

1 J. J. H. B. Sattler, A. M. Beale and B. M. Weckhuysen, *Phys. Chem. Chem. Phys.*, 2013, **15**, 12095–12103.

2 T. P. Van Swieten, A. Meijerink and F. T. Rabouw, *ACS Photonics*, 2022, **9**, 1366–1374.

3 P.-P. Knops-Gerrits, D. E. De Vos, E. J. P. Feijen and P. A. Jacobs, *Microporous Mater.*, 1997, **8**, 3–17.
